# Views on Researcher-Community Engagement in Autism Research in the United Kingdom: A Mixed-Methods Study

**DOI:** 10.1371/journal.pone.0109946

**Published:** 2014-10-10

**Authors:** Elizabeth Pellicano, Adam Dinsmore, Tony Charman

**Affiliations:** 1 Centre for Research in Autism and Education (CRAE), Department of Psychology and Human Development, Institute of Education, University of London, London, United Kingdom; 2 Wellcome Trust, Strategic Planning & Policy Unit, London, United Kingdom; 3 Department of Psychology, King's College London, Institute of Psychiatry, London, United Kingdom; University of Oxford, Kenya

## Abstract

There has been a substantial increase in research activity on autism during the past decade. Research into effective ways of responding to the immediate needs of autistic people is, however, less advanced, as are efforts at translating basic science research into service provision. Involving community members in research is one potential way of reducing this gap. This study therefore investigated the views of community involvement in autism research both from the perspectives of autism researchers and of community members, including autistic adults, family members and practitioners. Results from a large-scale questionnaire study (n = 1,516) showed that researchers perceive themselves to be engaged with the autism community but that community members, most notably autistic people and their families, did not share this view. Focus groups/interviews with 72 participants further identified the potential benefits and remaining challenges to involvement in research, especially regarding the distinct perspectives of different stakeholders. Researchers were skeptical about the possibilities of dramatically increasing community engagement, while community members themselves spoke about the challenges to fully understanding and influencing the research process. We suggest that the lack of a shared approach to community engagement in UK autism research represents a key roadblock to translational endeavors.

## Introduction

Basic scientific research is fundamental to improving human health. Yet the application of major scientific breakthroughs to where they are most needed, in clinics and communities, is often not swiftly forthcoming [1]. Translational research has therefore become a high priority for biomedical research policy around the world. Research centres have been established, grant programmes launched and scientists have been urged to think anew about the potential impact of their research. These initiatives have been motivated to accelerate “the translation of discoveries from basic laboratory and clinical science into benefits for human health [2] and to ensure public accountability for the investment in basic science. But whatever the precise driver, the core intention of translational research is clear: to close the gap between fundamental biomedical research and clinical, educational and related practice [3]–[5].

The potential benefits of translational research are nowhere greater than in the field of autism. There has been a dramatic increase in the recorded prevalence of autism in the past few decades [6]–[7]. Recent figures estimate that approximately 1% of the population in the United Kingdom has an autism spectrum condition [8]–[9], with similar estimates recorded in other parts of the world [10]. There has also been a dramatic expansion of research, particularly on neural and cognitive systems, genetics and other causal pathways both in the UK and abroad [11]–[12]. This surge in basic autism science promises to enhance the life chances of autistic people and their families. Yet the translational potential of this work remains unfulfilled, partly because only a minority of UK research funding is directed towards identifying effective treatments, interventions and services for individuals with autism [13]. As a result, the opportunities and life-chances for autistic people remain often severely limited in comparison with the non-autistic population. It has thus become imperative for autism researchers to reduce the translational gap.

In biomedical research more broadly, models of translational research have moved beyond a unidirectional bench-to-bedside approach to ones that also encourage *backwards translation*, with knowledge “from the bedside” informing research in the laboratory [5]. There is growing recognition, however, that even these bi-directional approaches can be overly simplistic; that the translational process is instead multidirectional and dynamic, including multiple points of knowledge exchange not only between researcher and practitioner communities but also, critically, between researcher and “patient” communities. That appears to be rarely the case in practice, however. Despite good intentions, in many current models [14] engagement of stakeholders is usually restricted to the dissemination and implementation phases – the very ends of the “translational pipeline”. Yet it is plausibly argued that translation will only be successful when scientific discoveries are more thoroughly *relevant* to “patients” and communities, are sufficiently *tailored* to the realities of their everyday lives and are consistent with their values [15]–[18].

In response, there has been a deliberate expansion of public participation across the UK's National Health Service (www.invo.org.uk), where health and social care researchers are encouraged to involve “patients” and members of the public as partners in the research process – working actively *with* community members rather than *on* or *for* them [19]. Such community engagement might be thought to be particularly important with regard to autism, given the long history of controversial claims by autism scientists – from “refrigerator mothers” [20] to the vaccine furore [21] – and the growing distrust of mainstream autism science by autistic self-advocates [22]–[24]. This entails that efforts to engage the broader autism community have advantages beyond any practical use they may or may not have in directly translating research findings into concrete changes in clinical and other practice. Engagement can potentially help overcome distrust between professionals and others, can strengthen the self-esteem and self-respect of researched communities and can help ensure that the research process properly responds to the interests and entitlements of autistic people [25]–[26].

Despite the potential importance of such efforts, almost nothing is formally known about the extent to which the autism community is engaged in research, beyond being involved as ‘subjects’. We simply do not know how commonplace formal or informal engagement practices are in autism research and whether a lack of community engagement represents a barrier to the translational endeavour. This study aimed to address these issues by asking both researchers and members of the autism community about their experiences of engagement in research. The autism community, however, is not homogenous and seldom speaks with a singular voice [25]. We therefore also examined potential differences in the degree and nature of the engagement experiences of distinct groups within the autism community. Rather than the more commonly used terms such as “patients”, “service user” or “stakeholder”, we use the term “autism community” to reflect the varied nature of this group, which includes those who are autistic themselves and those who care for, or who work with, children, young people and adults on the autism spectrum.

## Method

### Focus groups and interviews

Seventy-two participants took part in 11 focus groups and 10 individual interviews, including 14 autistic adults (2 female), 27 parents of autistic children (all mothers), 20 practitioners (18 female; 2 speech and language therapists; 16 teachers, 2 educational psychologists), 11 autism researchers (5 female; 6 early career researchers). Of the mothers, their children ranged in age from 5 to 19 years and also ranged in ability from those who had limited spoken language (n = 10) to those who they considered to be cognitively able or “high functioning” (n = 27). These groups were selected because they each held a “stake” in autism research. Autistic adults, parents and practitioners were recruited through community contacts across the UK. Researchers were recruited through personal contacts.

The focus groups and semi-structured interviews followed the same format. Discussion focused on participants' perceptions of current UK autism research and their priorities for future research (analysis of these data are presented in [13]). Towards the end of these discussions, we elicited participants' views and perspectives about the degree and nature of the autism community's engagement in research. Specifically, we asked about their experiences of being involved in research (or, in the case of researchers, involving the autism community in their research) and how they would like to be involved in the future.

Each focus group was kept exclusive (e.g., autistic adults only) and was conducted face-to-face in a location convenient for participants. Groups were led by a facilitator, who from time-to-time summarised the key points to the group and confirmed the interpretation of comments. Focus groups ranged from 44 to 124 mins (M = 93 mins). Interviews were conducted either face-to-face (n = 4), over Skype (n = 2) or on the telephone (n = 4), and lasted between 32 and 104 mins (M = 51 mins).

Where possible, focus groups/interviews were audiotaped and subsequently transcribed and analysed using the NVivo software package (Version 9). The resulting data were analysed using thematic analysis [27]. We adopted an inductive approach, providing descriptive overviews of the key features of the semantic content of data within an essentialist framework. Two of the authors (LP and AD) independently familiarised themselves with the data, meeting regularly to discuss preliminary themes and make a list of provisional codes. Each author then independently applied an exhaustive list of codes to the transcript of each interview/focus group. The authors met several times to review the results, resolve discrepancies and decide how the codes could be collapsed into themes and subthemes. All authors approached the coding and discussions from the perspective of autism scientists with an interest in public and community engagement in research.

### Online questionnaire

To reach a larger sample of the UK's autism community, we then developed an online questionnaire to gauge people's experiences of autism research in the UK. To encourage participation, this questionnaire was brief (11 questions), following a very similar structure to the focus groups/interviews. The design of the questionnaire was informed by the results of the focus groups/interviews, yielding changes to specific wording of items and the inclusion of the category ‘dissemination’ in our levels of engagement question (see below). This latter modification was in response to parents' wide-ranging experiences of hearing about autism research being conducted in the UK.

The questionnaire began with a series of background items (UK resident, primary interest in autism research, age, gender) followed by questions relating to participants' research priorities and their perspectives on the pattern of UK funding for autism research [13]. The final three questions related to the degree of engagement in research between the autism/research communities. Specifically, we asked participants (1) to indicate how often they had experienced three levels of ‘engagement’ (public dissemination, dialogue and partnership) between autism researchers and the broader autism community (on a 5-point scale from ‘very rarely’ (score of 1) to ‘very frequently’ (score of 5)), (2) to rate how satisfied they were with the level of engagement they had experienced (on a 5-point scale from ‘very dissatisfied’ (score of 1) to ‘very satisfied’ (score of 5)), and (3) to provide a reason for their stated level of satisfaction (open comment).

Respondents were told that engagement between researchers and the broader autism community could occur in a variety of ways including through:

1. *Dissemination*, where researchers provide information about the results of completed research through newsletters, online blogs, public events, etc.
2. *Dialogue*, where researchers communicate directly or consult with members of the autism community for their views about the research being conducted.
3. *Partnership*, where researchers and members of the autism community collaborate, as partners, in the research process, working together to set research goals and coming up with ways of realising them.

Our three levels of engagement differed somewhat from those advocated by INVOLVE (which include (1) consultation, (2) collaboration, and (3) lay control; see www.invo.org.uk and [28]) because (a) our initial results from the focus groups suggested that several community members had no previous experience of engaging in autism research (see below) and (b) instances of user-controlled autism research are extremely rare. Our levels of engagement therefore might be conceived of as lower down the rungs of a ‘ladder of participation’ [29] than those of INVOLVE but nevertheless reflected the current range of engagement activities in this particular field of research.

Participants were recruited through extensive community canvassing, including through autistic organisations, parent advocacy groups, practitioner and researcher networks, and via social media (Twitter, Facebook) and online fora. 1,632 people completed the engagement items of the questionnaire. To facilitate comparisons with the focus groups/interviews, analysis focused upon the 1,516 respondents aged 18 and over who could be divided into four key stakeholder groups: autistic adults, immediate family members, practitioners and researchers (see Table 1). The remaining 107 participants, who had labeled themselves as ‘other’ (e.g., student, “interested in autism”), were not considered further. For parents and carers (n = 825), the mean age of their child with autism was 13.4 years (SD = 9.0; range  =  2–57; 142 females) and, for sons, daughters or siblings (n = 24), the mean age of their autistic family member was 27.1 years (SD = 16.3; range = 4–65; 6 females).

**Table 1:** Descriptives for respondents to the online survey for each of the four key stakeholder groups (total n = 1516).

	Autistic^a^ person (n = 122)	Immediate family member (n = 849)	Professional (n = 426)	Researcher (n = 119)
Chronological age				
M (SD)	39.4 (12.9)	45.1 (9.8)	42.2 (11.8)	40.6 (13.8)
Range	18–72	18–83	21–70	22–87
Gender				
Female	56	765	350	81
Male	60	83	74	38
Other/would rather not say	6	1	2	1

Note: ^a^ The term “autistic person” is the preferred language of many people on the spectrum [50]. In this paper, we use this term as well as person-first language to respect the wishes of all individuals on the spectrum.

To ensure that our study was accessible as possible, autistic adults were invited to take part in the study using a range of formats (focus group, individual face-to-face interview, telephone/Skype interview and email); both the questions for discussion and the survey questions were offered to autistic participants so that they could review them in advance, if required; and two autistic adults acted as pilot participants and worked with the research team to make sure that discussion and survey items were comprehensible and easy to respond to.

### Ethics Statement

Ethical approval for this study was granted by the Faculty of Policy and Society's Research Ethics Committee at the Institute of Education, University of London. All participants gave written informed consent prior to participation and all data were collected anonymously.

## Results

### Focus groups/interviews: Researcher views

#### Uncertainty towards community involvement

**Figure 1:**
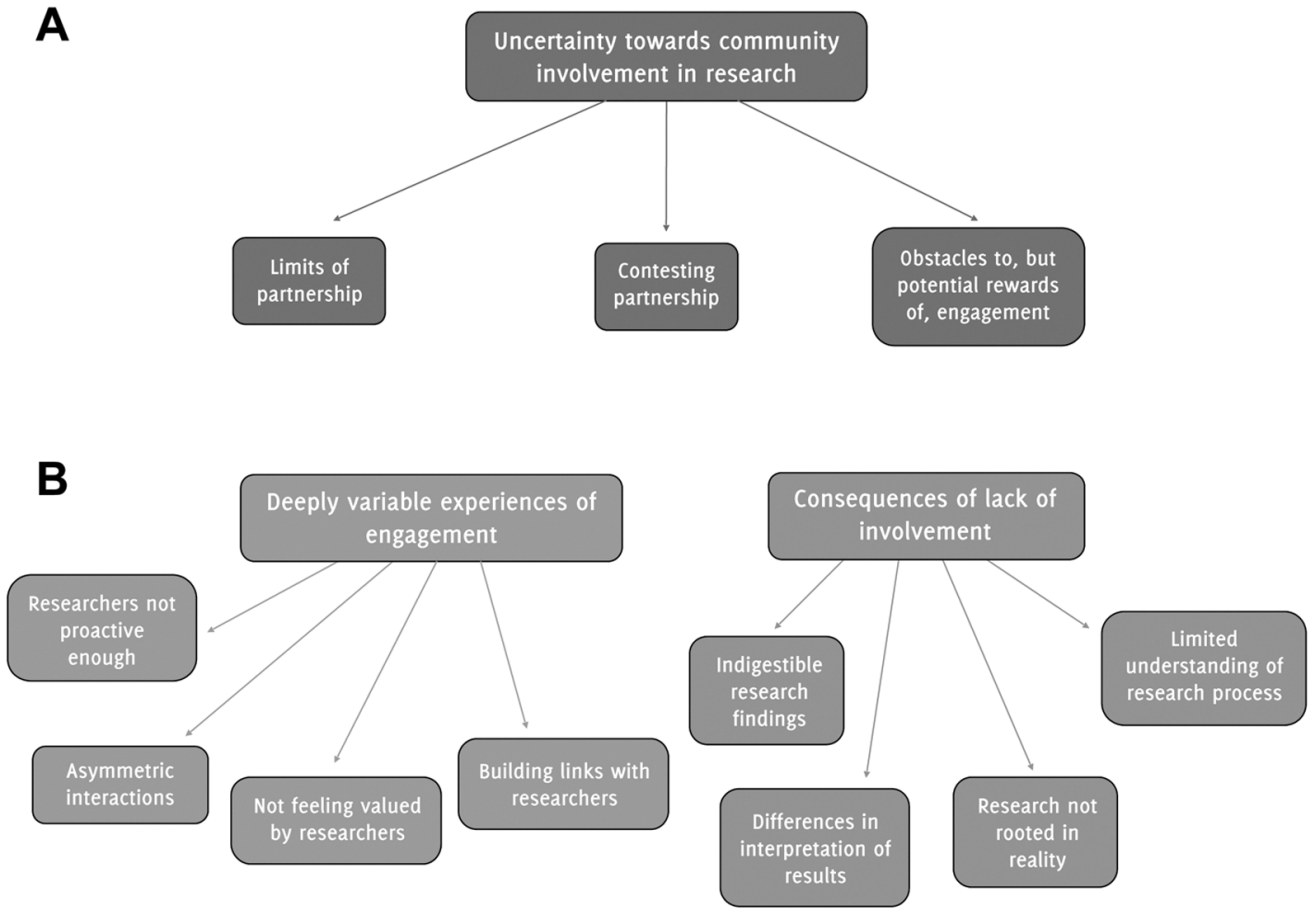
Researchers' (A) and community members' (B) perspectives on involvement in research: themes and subthemes.

The first subtheme related to the *limits of involvement* (see Figure 1A). For some researchers there was a strong sense of the need for real participation in research: “we have to involve them from the very start, in helping to define and shape the research”. Others felt that people making judgments about research and research funding “have to be other scientists” but that the viewpoints of different stakeholders should nevertheless be consulted: “I don't think that we want it to, the decision making, to move away from the scientists but I think they should at least get input from relevant stakeholders”. Others still were wary about involving autistic people and their families in decisions about research because (a) they might not be the appropriate people to decide what and how issues should be researched and (b) it risks “politicizing” scientific issues.

The second subtheme was *contesting partnership*. While there was consensus that involving members of the autism community as partners in research was not commonplace in autism research, researchers nevertheless differed in what they meant by “partnership”. Some researchers understood it as members of the autism community being involved in priority-setting exercises, while others talked of such members “contributing feedback” and “being on steering groups”. There were also suggestions that the nature and degree of involvement depended on the type of research being conducted: “People are much more likely to be better at involvement or engagement with people when there's a very direct patient interaction … whereas if you're dealing with anonymized urine samples or DNA samples then you're less likely to directly try and engage with stakeholders.”

The final subtheme related to the *obstacles to, but potential rewards of, community engagement*. Some researchers acknowledged the potential costs of community involvement, including the considerable time, money and effort spent in building and maintaining partnerships. Researchers were also concerned about the diversity of views within the autism community and the unlikelihood of any disputes being resolved. Others noted the potential benefits to involvement, including increased awareness of the research process by autistic people, families and practitioners and “a better chance that the research will get translated into practice”.

### Focus groups/interviews: Community views

The majority of autistic adults, parents of children with autism and professionals wanted to be more involved in the research process. The two themes identified related to their *deeply variable experiences of involvement* and the *consequences of lack of involvement* (see Figure 1B).

#### Deeply variable experiences of involvement

The nature of participants' experience of involvement in research varied widely but, overall, community members felt that *researchers aren't proactive enough*. Some parents, particularly those whose children had limited spoken communication, commented that they had never been approached by researchers to be part of research: “So I just find it really weird that, as a parent, you aren't approached about doing research, or providing information, that kind of thing, you have to go out and find it yourself.” These parents were perplexed that researchers were not “utilizing families” who were “waiting to be tapped into”. Practitioners noted the challenges of parental involvement, however, due to the busy nature of their lives: “it is often difficult to round up [parents] and get them involved”.

Parents and autistic adults with some experience of research emphasized the largely *asymmetric interactions with researchers*. Parents in particular highlighted that they “never get to find out the outcome.” One mother said, “I get a lot of asking for help. And then a bit less [contact] when something's going on, and hardly any results!” They wanted their involvement as participants to be valued by researchers. Autistic adults' reports were more negative, noting “sometimes we are a bit like monkeys in a zoo.” They spoke of the lack of reciprocity in their interactions with researchers: “they only want us as guinea pigs; that's the only contact they want with us.”

Autistic adults and parents also reported *not feeling valued by researchers*. Some participants were genuinely unaware that they could have a say in research while others were skeptical of the possibility of actually influencing research: “It's like not anybody is interested” (parent); “Whatever we say, is that really going to influence anyone?” (autistic adult). Some autistic adults reported that even when they had a voice, it was not listened to: “I just think sometimes when we say something we just sort of throw a spanner in the works, it doesn't suit their sort of agenda.” Others noted experiences of paternalism: “That's the danger of when someone knows you have a diagnosis, because there suddenly seems to be some sort of ascendency process that goes on and suddenly they have the right then to talk down to you, because you've got a label.” These adults stressed the need for researchers to value their expertise (of being autistic: “You have your area of expertise, which is not mine, and we have our area of expertise; you have to look at us on a similar level”) and for their involvement in research to extend beyond mere tokenism. Instead, they wanted to see more autistic people involved in research projects because “a lot of things aren't done with the autistic subjective kind of values and wants in mind.”

Practitioners were more positive about *building links with researchers* than autistic adults and family members. They highlighted the important links being built between researchers and their schools or clinics: “It's good that the people on the ground are trying to help support academia to come up with questions that are actually pertinent to what we want to find out about.”

#### Consequences of lack of involvement in research

Parents and autistic adults who had been involved as participants in research generally felt that *research findings were indigestible*. They emphasized their interest in research but also noted that they “haven't got the time to read lots of information, absorb it, react to it, go and search for something that links to it” (parent). Many commented that the information they received was not written in a “user-friendly” way, being full of scientific jargon: “when I read stuff that neurotypicals write about autism, for me it just reads like gobbledygook, it just doesn't make any sense” (autistic adult). They suggested that research findings “need to be simplified for them to understand what researchers are actually trying to put across” and needed to be targeted specifically at them, “coming through my door”, rather than having to have to find it themselves.

Parents and practitioners spoke of the disconnect between the research that goes on and that research that would actually make a difference to their lives or the lives of those they work with: “Researchers are not interested in social issues or anything like that, all they are interested is in medical issues” (parent). Parents reported feeling “jaded” and “cynical” about “pointless” research that is so far removed from their everyday experiences of autism. One mother said, “I fill in all these questionnaires and do everything I can to help and there's a very nice paper at the end with nice results and it's like “great”. But when it comes down to it, it's not real life. It's always missing the next step – great you've done this research, you've listened to my views, you've asked for them I've given them, but now do something with it.” Other parents felt as if the people making the decisions about research have probably “never been anywhere near someone with autism.” One mother said, “I think [contact with autistic people] would benefit the researchers, because they are kind of getting first hand experience from the people that are living with the children with autism … that I think would help towards the research because it's giving them an idea of what's really happening every single day.”

Some parents also felt frustrated with the lack of certainty that research provides; that there were no clear-cut answers or not enough research on the topics about which they needed answers. Their observations suggested that the lack of community involvement potentially resulted in a *limited understanding of the research process*: “I found as well that a lot of the researchers kind of contradict themselves, like one researcher will stand up and sort of say if your child goes on this gluten free diet it will better them, and improve this etc., and then you hear another researcher say no, that's rubbish, absolute rubbish.”

Autistic adults specifically spoke of their expertise (of being autistic) and the benefits that an insider autistic view would bring to research. They felt that the lack of community involvement in research led to *differences in interpretation*: “I feel that whoever's doing research is coming from a certain perspective, and you are starting off with an assumption that that person's disabled, and then you are looking at the research on the basis that we are disabled, like a rat in a cage, and if you do research like that you are probably going to end up very far, you know, confirming your own suspicions at the beginning.” One autistic adult suggested that, “having more autistic researchers or employing people on teams might guard against the possibility of misinterpretation [of findings].”

### Online questionnaire: Frequency ratings

Descriptive statistics for respondents' frequency ratings of engagement are shown in Table 2. With regards to *dissemination*, autistic adults, family members and practitioners reported that they had experienced dissemination of research ‘occasionally’ (mode score of ‘3’), while researchers reported ‘frequently’ (mode score of ‘4’) participating in dissemination with the broader autism community. With respect to *dialogue*, autistic adults and practitioners indicated that they ‘occasionally’ experienced dialogue with researchers but family members said that they ‘very rarely’ (mode score of ‘1’) experienced this sort of engagement. Researchers felt, however, that they ‘frequently’ participated in a dialogue with members of the autism community. All of the community groups stated that they had ‘very rarely’ (mode score of ‘1’) experienced *partnership* with researchers while researchers felt that they were ‘occasionally’ (mode score of ‘3’) involved in partnerships with autism community members.

**Table 2:** Respondents' mean frequency ratings for their experiences of each type of researcher-community engagement.

	Type of Engagement		
	Dissemination	Dialogue	Partnership
	M (SD)	M (SD)	M (SD)
	Range	Range	Range
Autistic adults	2.73 (1.3)	2.50 (1.2)	2.03 (1.2)
(n = 122)	1–5	1–5	1–5
Immediate family members	2.69 (1.3)	2.11 (1.08)	1.84 (1.0)
(n = 849)	1–5	1–5	1–5
Practitioners	3.12 (1.2)	2.42 (1.12)	2.16 (1.1)
(n = 426)	1–5	1–5	1–5
Autism researchers	3.48 (.96)	3.50 (.98)	2.82 (1.1)
(n = 119)	1–5	1–5	1–5

Note: Lower values reflect reduced frequency of involvement in research.

One-way ANOVAs on participants' mean ratings (see Table 2) with group as a factor (autistic adults, family members, practitioners, researchers) revealed significant group differences for dissemination, F(3, 1,515) = 22.69, p<.001, dialogue, F(3, 1,515) = 58.65, p<.001, and partnership, F(3, 1,515) = 33.28, p<.001. Post-hoc tests (Tukey's HSD) revealed the source of these differences. Note that because of the relatively large number of comparisons conducted, results are not reported as significant unless they reached a p value of less than .01.

Overall, researchers reported being engaged in dissemination, dialogue and partnership significantly more frequently than autistic adults, family members and practitioners reported experiencing such engagement (all ps<.01). There were also some subtle differences between community members' ratings. Autistic adults and family members reported being less involved in dissemination than practitioners (both ps<.005). Family members' mean ratings were significantly lower for dialogue than both autistic adults and practitioners (both ps<.005) but the latter two groups did not differ (p = .95). Finally, family members' ratings of the frequency with which they experienced partnerships with researchers were significantly lower (i.e., less frequent) than practitioners (p<.001) but not autistic adults (p = .22). Autistic adults' and practitioners' ratings did not differ (p = .64).

**Figure 2:**
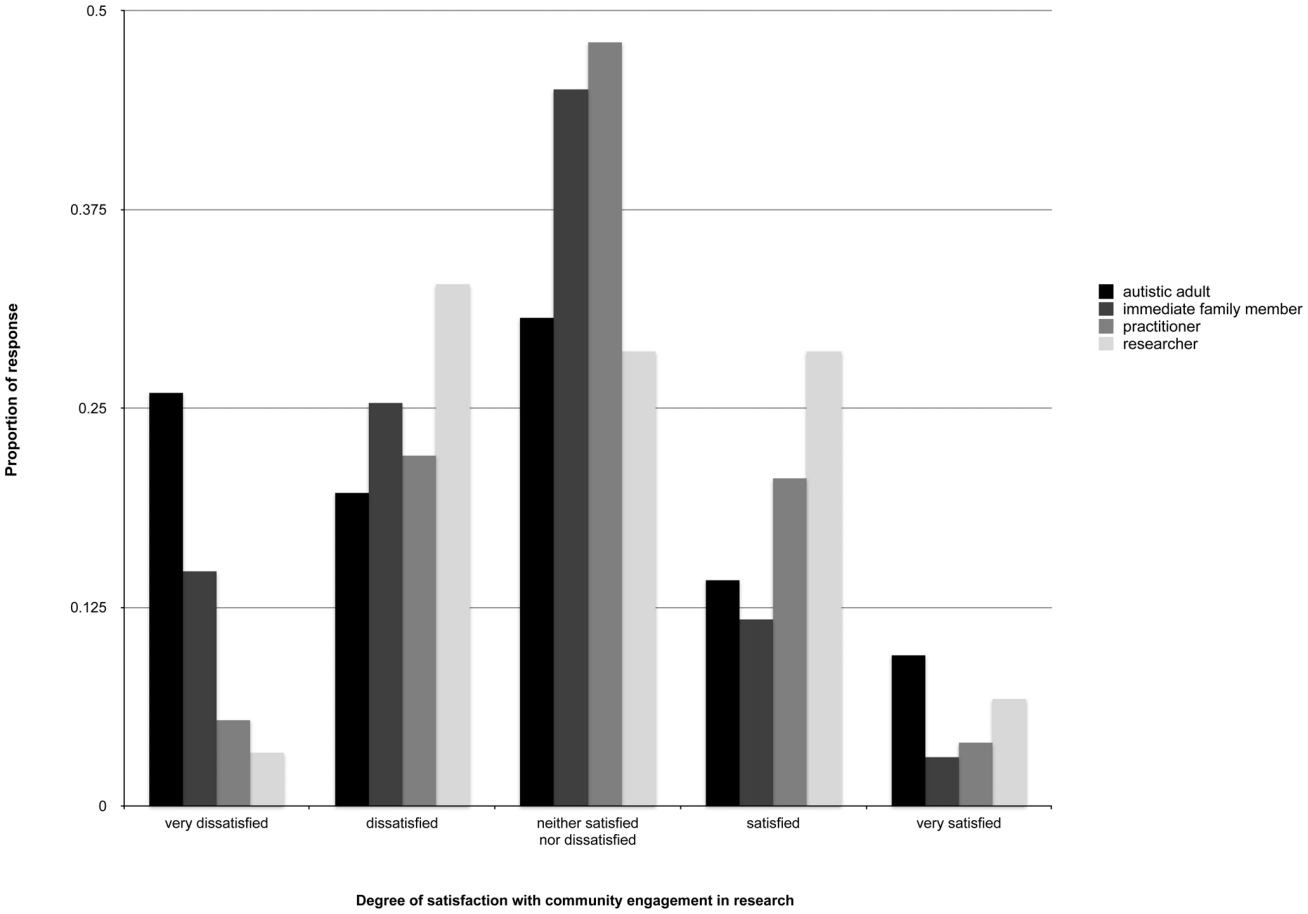
The proportion of responses for each stakeholder group with regards to their degree of satisfaction with community involvement in research.

We then examined the degree to which researchers and non-researchers were satisfied with this level of engagement. A one-way ANOVA on the mean ratings confirmed that the distribution of responses were slightly skewed towards ‘dissatisfied’ for autistic adults and family members but less so for practitioners and researchers (see Figure 2). There was a main effect of group, F(3, 1,515) = 14.16, p<.001. Researchers' (M = 3.02; SD = 1.01) and practitioners' (M = 2.96; SD = .90) ratings were significantly higher (reflecting greater satisfaction) than both autistic adults (M = 2.60; SD = 1.26) and family members (M = 2.63; SD = .97) (both ps<.005). There were no other group differences (ps>.90).

### Open question: Researcher views

Survey respondents were also asked to specify their reasons for their level of satisfaction with engagement in research (852 responses). Of the 63 researchers who responded to this question (see Table 3 for themes and corresponding quotes), several noted the importance of involving the autism community in the research process: “The perspectives of the population being researched should always be the first starting point.” All other comments centred on the *invitation to engage* and the *barriers to engagement* (see Table 3). Several researchers expressed how receptive and supportive the broader autism community had generally been to hearing about, or requests to take part in, research. Others' experiences were less positive, however, noting that involvement often amounted to a “tick-box exercise”. With regards to the potential barriers of involvement, some researchers pointed to a lack of a singular voice within the autism community, while others noted that the communication difficulties experienced by autistic people posed considerable problems for wider involvement in research.

**Table 3:** Themes identified from open question in online questionnaire by autism researchers (n = 63).

Themes	Subtheme	Example quotes
*Invitation to engage*	Positive community attitudes towards research	“The public are very interested to hear about autism research.” “Given the huge need for help and support for persons with autism, which is alas, often unmet, the public are very open and willing to take part in research that can go any ways towards this goal.”
	Scepticism toward involvement	“I feel that it can often be tokenistic, i.e. asking the same old panel of people with autism to contribute to policy, practice and decision making almost to “tick the box” to say that people with autism have been involved.”
*Barriers to engagement*	No singular voice	“The experiences of individuals with autism and their families are many and varied. Sometimes the most vocal individuals have a completely different experience/agenda than some of the most vulnerable people we engage.”
		“Attempts at engagement are very quickly dominated by more able people and people with additional mild to moderate learning disabilities, who could express their views with support, become swamped.”
		“Autism charities and groups are disparate and fractious.”
	Autistic features make involvement difficult	“Some of the challenges people with autism may face make the interactions quite difficult - trouble taking on board another person's point of view, commenting in a sensitive way that does not cause offense, etc. I would favour more partnership, but very different goals and methods of interaction make this a formidable challenge.”
		“It can often be difficult to work with people with autism as their viewpoints may be held very firmly and a ‘black and white’ thinking style can be a challenge.”
		“Due to the nature of autism, inclusion in group discussions/debates and decision making is difficult and time consuming (therefore expensive). I do feel these factors influence the true involvement of people with autism in research.”

### Open question: Community views

Comments from community members focused on similar themes, including their *experiences of engagement* in research and *barriers to engagement* (see Table 4). With regards to members' experiences of involvement, a common subtheme was the lack of awareness of the research being conducted. All three groups reported receiving few approaches from researchers to participate actively in research and there was uncertainty surrounding how they might come to know about such opportunities. Of those that had participated in research, many community members linked their level of satisfaction with engagement to researchers' attitudes. Respectful attitudes among researchers were often cited for high levels of satisfaction, although community members' experiences were not always so positive, with some highlighting a lack of interest in autistic adults' or practitioners' experience and expertise. Other dissatisfied community members focused on the nature of the often one-sided interactions with researchers. They reported being frustrated with the little or no feedback provided by researchers following participation.

**Table 4:** Themes identified from open question in online questionnaire by autistic adults (n = 94), immediate family members (n = 476) and practitioners (n = 219).

Themes	Subtheme	Example quotes
*Experiences of engagement*	Lack of awareness about research	“This is the first time I have heard of any research.” *Autistic adult*
		“I have never had any engagement.” *Autistic adult*
		“The research is conducted by, in the main, neurotypical researchers looking at, not surprisingly, neurotypically framed problems and questions. There are too many assumptions about what it is like to be autistic, what autistic people want and possibly most important, what autistic people need.” *Autistic adult*
		“I feel isolated from any sort of research. I have very little knowledge of any research that may be going on or what its purpose is. Is it purely academic? Does it have practical applications, and if so, how? ” *Parent*
		“There is just so little research that I'm aware of. Once diagnosed, you're left to get on with it unless you have the time and inclination to get involved in support groups.” *Parent*
		“I have never been approached or asked to take part in research although I would be interested to do so.” *Practitioner*
	Experiences depend on researcher attitudes	“The autism researchers that I dealt with personally were always interested in what I had to say, as well as my well-being.” *Autistic adult*
		“I find a majority of autism researchers I have managed to speak to unapproachable and more concerned with engaging in academic debates about autism as opposed to speaking to autistics.” *Autistic adult*
		“Those I have come into contact with have had a genuine interest and concern for people with autism.” *Parent*
		“Some of the researchers have been informative and collaborative, others have no interest in what practitioners need or have to share” *Practitioner*
	Asymmetric interactions	“Despite most research projects claiming that information about the results of these projects will be sent to me upon their completion, I very rarely, if ever, receive any such information. It almost feels like they've got what they need from me by this point, so they don't really need to contact me again with the results, as they won't get anything back by doing so.” *Autistic adult*
		“Researchers are more keen on collecting data, but not providing results” *Autistic adult*
		“I would like more detail of the results of research, particularly where I have given time and effort to helping with it. I sometimes only get a very short summary of the research, and often I would welcome more detail.” *Parent*
		“Often have not had feedback from the results of a research project and often left thinking… and so? What does that matter, what happens next/what difference does that make?” *Practitioner*
*Barriers to engagement*	Lack of opportunity to get involved	“I've been turned away from a few studies for being female.” *Autistic adults*
		“In my experience researchers are only interested in helping those at the more able end of the spectrum.” *Parent*
		“I do get asked for help in research issues but as I work full time and my son is ASD I don't have the time to do them. My son is at respite today and I am having a day off which is the only reason I've been able to complete this.” *Parent*
		“Carers don't have much time or energy left over for things that don't directly affect our ability to deal with the day to day issues, however much we want access to latest thinking.” *Parent*
		“As a practitioner in the public sector I am overworked and often not able to find time to actively engage with current research, though I like to stay informed on research reports” *Practitioner*
	Skeptical about researcher intentions	“Being used by researchers to further their own career.” *Autistic adult*
		“Most autism researchers are engaged in research that I find unacceptable, i.e. looking for ‘cures’ or which seem to ‘objectify’ autistic people as odd or freaks or severely flawed.” *Autistic adult*
		“I don't think many researchers feel they can talk to autistic people as if they matter, they're too busy studying them like specimens or looking for a ‘cure’.” *Practitioner*
		“There are pockets of joined up working which are excellent but there are also huge silos within the world of autism research.” *Practitioner*
	Absence of accessible, user-friendly research	“Lots of research is not in the public domain and requires subscriptions.” *Autistic adult*
		“I have only been involved because I have been proactive myself. Families have lots of information about living with the effects of Autism yet researchers don't seem to be tapping in to this resource.” *Parent*
		“It's hard to find out about the research and about what's available publicly. Useful info and links tend to come via word of mouth - other routes are typically time-consuming, material often not user-friendly.” *Parent*
		“It should be easier to access research papers published in journals. Researchers should be prepared to publish pdfs of their own research papers, since members of the autism community often do not have access to journal subscriptions.” *Parent*
		“We live in a society where it can be hard to be sure of who is a credible source of information/opinion when online, either via blogs or social networking etc. Open access to journals may increase the public's opportunity to find out about the latest autism research from a reliable source.” *Practitioner*
		“As an autism professional I have to seek out any information regarding research myself, very rarely is it in a format that is easy to comprehend for a non academic/researcher.” *Practitioner*
	Research topics not rooted in reality	“Feels like researchers are working in their own world, with little direct engagement with us. Probably they are following the funding and have become isolated from the practical issues people face.” *Parent*
		“Those of us who live and breathe autism and who have to manage daily to support our children can feel left out of the debate. Researchers will look for something of interest TO THEM - and not necessarily useful to the autism community in any practical sense.” *Parent*
		“I feel most UK researchers operating from ivory towers with very little contact with real autism.” *Parent*
		“Far too interested in causes and cures with intellectual understanding only and no practical application.” *Practitioner*
		“It's never about the issues that persons with autism face. It's too airy, too detached from practical application and frankly a waste of time and money. Researchers need to be helping these people, not simply writing papers about them.” *Practitioner*

The second theme again centred on *barriers to engagement* in research. For autistic adults and parents, these barriers included their own or their autistic relative's age, gender, and level of functioning; research often excluded adults, especially older adults, girls and women, and those with additional (and severe) intellectual disabilities. Parents also cited the prohibitive time demands of caring for their child with autism and practitioners spoke of difficulties finding the time for involvement in research in their already busy lives. Finally, for some respondents, their experiences of involvement had led to some scepticism about research. Many autistic respondents credited their lack of satisfaction to what they described as “dehumanizing” interactions (“being treated like guinea pigs”) and the predominance of a neurotypical outlook (i.e., one focused on cure and prevention of autism) among researchers.

Some autistic adults, parents and practitioners felt that they had limited access to research and its findings, and were especially critical of the prevalence of prohibitive paywalls and journal subscription requirements. Both parents and practitioners were critical of the language used in research reports, which made it inaccessible to lay audiences, and which caused them to feel “swamped” and “bombarded” with information they did not understand. Community members also commented on researchers' priorities for autism research, which seemed to conflict with their own. They were more likely to feel satisfied by their level of engagement in research if the projects in which they participated had explicit, practical applications. Some members, especially parents and practitioners, reported feeling “let down” by research that had no bearing on their everyday lives.

## Discussion

Within traditional models of research, researchers design and implement their studies, and interpret and disseminate the findings, only seeking interaction with the community during the recruitment and participation stages. Yet the need to speed up the translation of basic science findings into practical applications has prompted calls for greater participation in the research process by communities across health-related research [4] (www.invo.org.uk). This study is the first to investigate the degree and nature of community involvement in the field of autism research both from the perspectives of researchers and of members of the autism community. We sought to understand current engagement practices and identify potential barriers and opportunities to engagement in research and, ultimately, to the translational endeavour.

There were competing views between stakeholder groups regarding the degree of engagement in current UK autism research. Overall, researchers perceived themselves to be engaged with the autism community – in terms of the extent to which they disseminated their findings, consulted the autism community and developed partnerships with its members – but other stakeholders, most notably autistic people and their families, did not share this view to the same degree. Practitioners were more likely to have engaged with researchers either through dialogue or through the building of research-practice networks and were generally satisfied with this degree of engagement. Given that models of translational research have traditionally emphasised knowledge exchange between researchers and practitioners, this finding is perhaps not surprising. Autistic adults and particularly family members were less satisfied with their involvement, however, which ranged widely from non-participation to acting as consultants in research. It is true, of course, that these results may not be representative of the entire autism community – particularly those who cannot communicate well enough to advocate for themselves – but the fact that the findings from the online questionnaire mirrored the findings in the focus groups/interviews give us reason to believe this dissatisfaction is widely shared.

This relative lack of satisfaction by certain community members might be driven by unrealistic expectations of research, in which progress can be frustratingly slow, and for parents in particular, anxieties about the need for “quick fixes” and help in the here-and-now. The challenges surrounding the communication of results and the realities of research to the public are not specific to autism research, but occur across science and humanities [30]. The core skill set of researchers – including experimental design, statistical analysis and communication of results to academic peers – does not involve mechanisms of outreach, especially with potentially vulnerable groups. This issue might well be a problem with researcher training or a lack of clear guidelines [31] but will nevertheless take some time to change.

Beyond better science communication, there were also conflicting views about the nature and extent of involvement in autism research by researchers themselves. Individual researchers had very different conceptions of what engagement in research does – and should – look like. Some researchers felt strongly that the autism community should be more involved in research, largely in identifying research priorities, which would increase participation and external validity. Others, however, were apprehensive about such involvement, perceiving it as a potential threat to scientific (internal) validity. Notably, all of our researcher respondents implicitly viewed the autism community as relatively passive in the research process, rather than actively involved in knowledge production. Indeed, *no* researcher suggested that community members should be co-producers of research, that is, that the balance of power with regards to key scientific decision-making processes (priority-setting, funding decisions, design, implementation, interpretation or dissemination) should be equal between researchers and members of the autism community, as it is in community-based participatory models of research [32]–[33], and none mentioned the possibility of user-controlled research (www.invo.org.uk).

This (implicit) resistance to engaging the autism community may be related to the largely negative descriptions of autistic adults' and family members' interactions with researchers. Family members felt disappointed and frustrated at being ‘mined’ for information and having little or no opportunity to learn about the resulting discoveries and what they might mean for them, while autistic adults reported feeling objectified (“we are a bit like monkeys in a zoo”) and their experiential expertise disregarded by researchers. This lack of reciprocity resulted in feelings of distrust and being less motivated to participate in future research.

This pattern is not unique to autism research. Other studies examining researchers' attitudes to user involvement in health research also report feelings of apprehension, particularly with regard to challenges to traditional knowledge production and acquisition [34]–[35]. Scientific research prizes itself for being impartial, falsifiable and rigorous. For some, the very involvement of those with a vested interest (e.g., patients) potentially introduces bias or reduces objectivity, and is thus seen as less valid and reliable.

Not only does this argument imply that researchers are bias-free (which is not the case; [36]–[37]), it also suggests that the only people permitted to make decisions about research are researchers themselves. Moreover, in some, possibly many, cases, the benefits of community involvement outweigh the (putative) risks. Community involvement in research theoretically increases the likelihood that research findings will be implemented in communities because their involvement ensures that (i) research findings and interventions are accessible, useful and sustainable, (ii) research addresses issues of real-life practical import, and (iii) services and interventions are tailored to fit the community's needs [14], [38]. Without such involvement, the research findings are at risk of being ‘lost in translation’.

Contemporary models of patient involvement have built upon Arnstein's “ladder of citizen participation” [29] in which the rungs of the ladder reflect increasing levels of participation and degree of citizen control in decision making ranging from *non-participation* or manipulation and therapy through to *tokenism*, including informing, consulting and placating, and to the higher rungs of *citizen power* via partnership, delegated power and citizen control. Current policy initiatives, including the UK's Department of Health INVOLVE initiative (www.invo.org.uk), aim to move patient involvement in research “further up the ladder”, increasing the degree of involvement – and decision-making power – in the research process. Critics have suggested that the higher rungs of the ladder may not be easily attainable in research because research is often researcher-led [39]. There is, however, a strong literature of successful participatory or emancipatory research with people with intellectual and learning disabilities [41]–[43] and there are rare exceptions in US autism research [33], [40], providing proof that partnership in research is at least sometimes possible and can be achieved in such a way as to accommodate the fact that the autism community is both diverse in its needs and is geographically widely dispersed. The current findings suggest, however, that the degree of community involvement in UK autism research remains close to the bottom of the ladder.

This lack of involvement in autism research might be one reason for the apparent mismatch between what is currently being researched in the UK and what community members want from such research. A recent survey of the state of current autism research in the UK found that the majority of funded research between 2007 and 2011 focused upon understanding the underlying biology and causes of autism (i.e., basic science) [13]. When consulted about the UK's research profile, there was overwhelming consensus from community members that the imbalance in current research must be addressed, with greater priority on research that has an immediate, practical impact on people's everyday lives [44]. Autistic people, their family members and practitioners are rarely actively engaged in the research process – in deciding how an issue is researched, how it becomes funded, who undertakes the research and so on. Developing ways to involve the autism community both in priority-setting exercises in specific areas and in the research process more broadly is one way to ensure that a greater portion of research has a direct and sustained impact on those who need it most.

How, then, can we support greater community involvement in research? There is no ‘one size fits all’ approach to community engagement. It will necessarily vary according to the research aims, the project, the target participants, the individual researcher(s) and so on. The process should therefore both be experimental and iterative. Researchers should be encouraged to innovate in a variety of ways as they develop more widespread mechanisms of engagement between researchers and the autism community, doing so in ways that seem suitable to the particular circumstances and which reflect continuing discussions and debates concerning the right way to represent autistic people and other community members. Researchers need to develop mutually supportive and respectful relationships with members of the autism community – relationships that are both intrinsically valuable and necessary for the transfer of research findings into practice [45]–[46].

Developing these research-community partnerships takes time, effort and often funding. Indeed, our researcher participants mentioned a lack of supportive infrastructures as a real barrier to community engagement. Others [35], [38] have highlighted many challenges, including lack of time, energy, and resources to build and sustain partnerships, limited funding mechanisms and institutional commitment, and lack of researcher training, especially regarding power-sharing arrangements, raising questions regarding whether such intensive community engagement is suitable for all research. Some of our participants suggested that the degree of engagement in research might differ depending on the area of research. While it is true that basic or laboratory-based scientists might be less likely to interact with, and thus engage, the autism community, or to perceive benefits from such engagement, this distance should not be a rationale for limiting engagement. Many aspects of genetic and biomarker research – the hallmarks of translational research – conducted in basic science laboratories carry complex social and ethical implications related to “risk” (of developing autism) and cure and prevention [25], [47]. These very issues are the ones that often provoke the most unease within the autism community and extra efforts must be taken to ensure that autistic people and their families are involved, not excluded, from basic science research [26]. Engaging with UK community members should be the first step in determining how and to what extent they might shape the research process.

Grant-giving bodies and government agencies should work towards developing supportive infrastructure, actively encouraging researcher-community dialogues at all stages of the translational process, and providing the necessary training. Some research should aim to involve partnerships between researchers and community members, which should be genuinely participatory and not just tokenistic, where autistic people and other key stakeholders are ‘co-producers’ of the research. Building such institutional mechanisms of engagement requires sustained effort. There are ways, however, in which researchers can act now to develop mutually supportive partnerships with the autism community. Researchers should actively promote their research, ensuring that it reaches community members in an accessible (i.e., jargon-free) manner. They should also listen to the views and perspectives of the autism community to understand – and value – what it is like to be autistic, to care for someone who is autistic, or to work with someone who is autistic – valuing their expertise and thus reducing the epistemological divide [35]. Similarly, the autism community should work towards increasing their ‘research literacy’, gaining a better understanding of research and the challenges involved.

### Conclusion

While the call for greater community involvement in research is not new [48], the current findings suggest that autism researchers have not readily embraced it. The lack of commitment to involving the community in research potentially presents a significant challenge for successful translational autism research. Ensuring that the advances in research impact upon those who need them most requires sustained engagement with the community at all stages of the translation process, however difficult that may seem – from establishing the research priorities and conducting the research, to disseminating and implementing the final products/intervention – while at the same time maintaining scientific rigor. Building and maintaining mutually supportive partnerships is one viable way of achieving this goal [49]. As these partnerships unfold, researchers will need to explore in detail people's experiences of them and, ultimately, to determine their impact.
